# Dysregulation of TCTP in Biological Processes and Diseases

**DOI:** 10.3390/cells9071632

**Published:** 2020-07-07

**Authors:** Ulrich-Axel Bommer, Adam Telerman

**Affiliations:** 1 School of Medicine, Faculty of Science, Medicine & Health, University of Wollongong, Wollongong, NSW 2522, Australia; 2 Institut Gustave Roussy, Unite Inserm U981, 94805 Villejuif, France

**Keywords:** TCTP (HRF, fortilin), growth and development, biological stress reactions, autophagy, regulation of protein synthesis, regulated protein degradation, cancer, cardiovascular diseases

## Abstract

Translationally controlled tumor protein (TCTP), also called histamine releasing factor (HRF) or fortilin, is a multifunctional protein present in almost all eukaryotic organisms. TCTP is involved in a range of basic cell biological processes, such as promotion of growth and development, or cellular defense in response to biological stresses. Cellular TCTP levels are highly regulated in response to a variety of physiological signals, and regulatory mechanism at various levels have been elucidated. Given the importance of TCTP in maintaining cellular homeostasis, it is not surprising that dysregulation of this protein is associated with a range of disease processes. Here, we review recent progress that has been made in the characterisation of the basic biological functions of TCTP, in the description of mechanisms involved in regulating its cellular levels and in the understanding of dysregulation of TCTP, as it occurs in disease processes such as cancer.

## 1. General Overview

TCTP (also referred to as: HRF, fortilin, P23; gene symbol tpt-1; in yeast: TMA19 or Mmi1) was first described almost 40 years ago as a growth factor-induced, translationally controlled protein in murine cell lines [1,2]. The elucidation of its biological importance was initially hampered by the fact that, based on its amino acid sequence, it is not related to any other known protein family. It took a considerable international research effort, spread over more than three decades, to piece together the puzzle and reveal our current picture of the biological function of TCTP and of its involvement in various diseases. Several review articles on TCTP/HRF/fortilin have been published previously, and the first ‘TCTP book’ appeared in 2017, a compilation of 16 individual review articles covering a wide range of topics relating to this multifunctional protein [3]. Since then, a considerable number of new papers reported various novel aspects on TCTP, and it is the aim of this review to summarise these more recent additions to the ‘TCTP story’. Being part of the Special Issue ‘Role of TCTP in Cell Biological and Disease Processes’ in *Cells*, our review will only briefly touch on those topics that are the subject of the other review articles within this Special Issue. However, we will discuss the research papers of the Special Issue, where appropriate, to put these into context.

## 2. Importance of TCTP in Core Cell Biological and Stress Reactions

The involvement of TCTP in a multitude of cell biological processes is reflected by the large number of interacting proteins. Compilations of the many interaction partners of TCTP have been published in the above-mentioned book [4,5,6]. A more recent study, using current proteomics approaches combined with in silico network and gene ontology enrichment analysis, identified 113 interacting proteins [7], which were allocated to the following gene network clusters: translation, stress response, cytoskeleton, signal transduction, proteasomes, nucleosomes and transcription, RNA binding and metabolic enzymes. These clusters can be roughly associated with the following three core biological processes. We will discuss recent advances on the participation of TCTP in these processes, as summarised in Table 1 below.

### 2.1. Growth and Developmental Processes

There is a considerable body of evidence for the involvement of TCTP in cell and organ growth, as well as in developmental processes, most aspects of which were reviewed in the following chapters of the ‘TCTP book’: cell cycle progression [5,8], early development [5,9], and organ growth and development [9,10,11]. Since then, several new studies have extended our insight into potential mechanisms, through which TCTP might participate in these processes.

Regarding cell cycle regulation in early development, Jeon et al. showed that TCTP regulates spindle assembly during postovulatory aging of mouse oocytes, thereby preventing deterioration of oocyte quality [12]. The targeting of yet another mechanism for cell cycle control by TCTP was revealed in a more recent paper [13]. The authors showed that TCTP interacts with CSN4, a subunit of the COP9 signalosome complex, which controls the G1/S transition of the cell cycle through regulating Cullin–Ring ubiquitin ligases. This mechanism is conserved between plants (*Arabidopsis*) and insects (*Drosophila*). The latter observation is consistent with another report showing that disruption of TCTP using CRISPR/Cas9 in the silkworm *Bombyx mori* resulted in developmental arrest and in subsequent lethality in the third instar larvae, caused by defects in proliferation of intestinal epithelial cells [14]. Similarly, a recent report demonstrated the importance of TCTP in stem cells of the midgut in *Drosophila* for tissue homeostasis and regeneration [15]. As potential mechanisms, the authors suggest the regulation of protein synthesis by TCTP (Section 2.2. below) and crosstalk with two important growth regulating signalling pathways.

The importance of TCTP for organ development was supported by a new report showing that TCTP promotes liver regeneration via mTORC2/Akt signalling [16], by studies on axon guidance [17,18,19] and on brain development [20]. Roque et al. demonstrated the role of TCTP for axon guidance and development in the visual system of *Xenopus laevis* [18] and the importance of its localised translational regulation in axonal growth cones [17]. A recent paper confirmed the importance of TCTP for general brain development in mice [20]. Conditional TCTP-knockout mice displayed retardation in brain development and died at the perinatal stage. An interesting case of the involvement of TCTP in organ development was reported for plants [21]. The authors demonstrated that in *Arabidopsis thaliana*, the mRNA (and protein) of the AtTCTP1 protein is transported over a long distance from scions into the roots, where it provides a signal to govern the formation of lateral roots. In contrast, the ‘endogenous’ AtTCTP1, locally produced in the root cells, drives the elongation of the roots. Thus, TCTP is a molecule crucial for establishing the root architecture of plants.

### 2.2. Regulation of Protein Synthesis and Degradation

At the beginning of this century, the association of TCTP with ribosomes [22] and the translational machinery [23] was identified in yeast. A more specific study demonstrated the interaction of TCTP with translation elongation factor eEF1A and its guanine nucleotide exchange factor (GEF), eEF1B [24]. Subsequently, the interaction of TCTP with elongation factors of the EF1 family and with other components of the translational apparatus was confirmed both in human cells [7,25] and in *Drosophila* [15]. Consistent with this is the observation that the genes of ribosomal proteins, elongation factors, and of TCTP all belong to the class of ‘TOP genes’, whose mRNAs have a common signature, the 5′-terminal oligo-pyrimidine tract (5′-TOP) [26], and are therefore translationally regulated [27].

Later, the interaction of TCTP with eEF1B was studied in more detail, using a range of structural methods [28]. This paper demonstrated that TCTP binds to the central acidic region of eEF1B and that in both these proteins the mutually interacting regions are highly conserved in evolution, thus representing the most conserved interaction of TCTP. This conclusion was also supported by the recently published solution structure of TCTP from a unicellular micro-alga [29].

The functional importance of TCTP in its interaction with translation elongation factor 1 and/or its GEFs still needs to be fully clarified. The initial observation was that, through binding to eEF1Bbeta, TCTP impaired the GDP-GTP exchange reaction on eEF1A, stabilising it in its GDP-bound form [24]. A variation of this mechanism was proposed very recently by a Japanese group interested in mechanisms involved in formation of neurofibromatosis type 1 (NF1)-associated tumors. Their work confirmed the interaction of TCTP with EF1A, as well as with its GEF complex consisting of EF1B, EF1G, and EF1D [7]. In addition, this paper showed that the interaction of TCTP with EF1A2, an isoform of EF1A preferentially expressed in neuronal tissue and skeletal muscle, is much stronger compared to that with the normal isoform, EF1A1. The authors concluded that, in NF1-associated tumor cells, TCTP binds to the GDP-bound form of EF1A2, thus preventing its dimerisation and inactivation. In this way, TCTP facilitates the binding of the GEF complex to GDP-bound EF1A2, promoting the GDP-GTP exchange reaction and recycling of EF1A2 [7].

Whether the net effect of TCTP on translation elongation is positive [7] or negative [24], both should result in a general regulation of protein synthesis. However, another well-documented example of translational control that targets the elongation cycle of protein synthesis has been shown to result in a selective translational advantage for specific mRNAs. Elongation factor 2 (EF2)-kinase phosphorylates EF2, thereby slowing down protein synthesis. This results in an increased expression of proteins implicated in cell migration and cancer cell metastasis [30]. It remains to be seen whether the effect of TCTP on EF1 activity may also lead to a preferential translation of certain mRNAs.

Two additional observations confirm the involvement of TCTP in the translational machinery, i.e., the recently reported interaction of TCTP with the receptor of activated protein kinase C (RACK1) [31] and the identification of TCTP as an mRNA-binding protein in HeLa cells (Supplementary Table S1 in [32]). These two observations may be related to each other, since RACK1 was identified as a ribosomal protein that is located close to the ribosomal mRNA entry site [33]. RACK1 serves as a ribosomal scaffolding protein that is involved in targeting the ribosome to various signalling modules and also to specific subcellular locations, such as focal adhesion sites [33].

Several examples of an involvement of TCTP in the regulated degradation of specific proteins have been reported. A review on this topic was published in the TCTP book [34]; here we will briefly summarise the relevant examples (see also Table 1 below):

1. TCTP stabilises the following client proteins by masking their ubiquitination sites, thereby preventing their proteasomal degradation. These are the anti-apoptotic protein Mcl-1 [35], the protein kinase Pim-3, a proto-oncogene [36], the antioxidant enzyme peroxiredoxin PRX1 [37], and the tobacco histidine kinase 1, NTHK1 [38]. This kinase represents the receptor for the phytohormone ethylene in plants. In this way, TCTP reduces the plant response to ethylene, which enhances plant growth and cell proliferation [38]. A specific case is the stabilisation of hypoxia-inducible factor HIF1α, where TCTP does not bind to HIF1α itself, but to the von-Hippel-Landau protein (VHL), which normally acts as E3-ligase for HIF1α. TCTP binding results in ubiquitination of VHL and in its own proteasomal degradation, preventing the degradation of HIF1α [39].

2. For other proteins, TCTP actively promotes degradation. For example, to induce degradation of the tumor suppressor protein p53, TCTP binds to p53-MDM2 complexes and inhibits MDM2 auto-ubiquitination, resulting in MDM2-mediated ubiquitination and degradation of p53 [40]. TCTP also promotes the degradation of cell cycle protein phosphatase Cdc25, which is important for an orderly mitotic exit. TCTP overexpression, as it occurs in hepatocellular carcinoma (HCC), induces ubiquitination and degradation of Cdc25, eventually resulting in chromosome missegregation [41].

3. Apart from regulating the degradation of specific proteins, in yeast, TCTP (TMA19; Mmi1) has also been shown to interact with proteasomal proteins [42] and to colocalise with the proteasome under heat stress conditions [43]. Yeast Mmi1 slightly inhibited proteasome activity, and was found to colocalise with two proteins of a de-ubiquitination complex in heat-stressed cells [43].

### 2.3. Biological Stress Reactions and Autophagy

The role of TCTP as an anti-apoptotic protein involved in protecting cells in a range of stress conditions (inclusive of DNA damage [44]) in mammalian cells [5,45], in plants [9], and in insects [10] has been reviewed in several articles of the TCTP book [3]. Here, we will just summarise the more recent contributions in this area.

Two recent papers demonstrated the importance of TCTP as a survival factor in mammalian organs. Cai et al. showed that TCTP plays a critical role for the survival of cardiomyocytes and has a protective function against drug-induced cardiac dysfunction in mice [46]. Another paper, published in this Special Issue in *Cells*, studied the importance of TCTP for the development of the central nervous system [20]. The authors generated mice that are disrupted in TCTP expression in neuronal and glial progenitor cells. These mice die at the perinatal stage, and they show slight abnormalities in early brain development that are associated with increased apoptosis, demonstrating that TCTP is a critical protein for cell survival during early neuronal and glial differentiation.

It was well established for quite some time that TCTP is involved in protecting cells in a wide range of stress conditions. However, up until recently, it was not known that it also plays a role in the ER-stress defense program, the unfolded protein response (UPR). This gap has been closed by a recent paper by Pinkaew et al. [47]. IRE1α is one of three main players in initiating the UPR; it has protein kinase and endonuclease activity and is ultimately responsible for the induction of apoptosis, once the protein overload in the ER becomes overwhelming. The authors showed that TCTP (fortilin) is able to bind to phosphorylated IRE1α, thereby preventing the activation of the JNK apoptosis pathway by IRE1α.

The importance of TCTP in biological stress reactions was also demonstrated in non-mammalian systems. De Carvalho et al. overexpressed TCTP from tomatoes in tobacco plants, which resulted in an increased growth rate and an improved performance under salt and osmotic stress conditions [48]. The transgenic plants displayed an increased expression of genes involved in photosynthesis, fatty acid metabolism, and water transport, and this was paralleled by an increased photosynthetic rate. On the other hand, genes involved in ethylene biosynthesis (a plant hormone) were down-regulated by TCTP overexpression. This is consistent with the observation that TCTP binds to the ethylene receptor in tobacco plants and reduces its response to ethylene [38]. Studying the role of TCTP in Trypanosomes, Jojic and co-workers reported that, in the bloodstream form of the parasite, depletion of TCTP expression resulted in a reduced growth rate and also in a slower recovery after heat stress [49]. The importance of TCTP in the development of resistance against the insecticide deltamethrin was investigated in Drosophila kc cells [50]. RNAi-knockdown and overexpression experiments confirmed that TCTP partially protects these cells against deltamethrin-induced cell death.

Over the past decade, autophagy has been recognised as a central biological process, essential for maintaining cellular homeostasis; but the involvement of TCTP in this pathway has only recently been investigated. In the ‘TCTP book’, this aspect of TCTP function has only briefly been touched on in two articles [5,34] and also here, we will not cover it in much detail, since another review article in this Special Issue discussed the role of TCTP and autophagy in relation to tumorigenesis [51].

So far, the effect of TCTP on autophagy has only been studied in three original reports (Table 1), with partially conflicting results. The earliest of these papers found that TCTP is one of five genes upregulated by artificial selection in the ovaries of domesticated vs. wild pigs [52]. The authors also observed that TCTP is located in the cytoplasm, in a pattern similar to the autophagy protein LC3. In COS-7 cells kept under normoxic conditions, TCTP knockdown resulted in enhanced AMPK activity and an increase in the levels of the LC3-II protein, whereas the opposite was true under hypoxic conditions. The observed effects could be reversed by re-introducing the TCTP gene into the cells via a lentivirus construct. TCTP was also found to interact with the ATG16 complex of autophagic proteins. The authors concluded that TCTP positively regulates autophagy via the AMPK/mTORC1 pathway. Another, more recent paper arrived at a different conclusion i.e., that in HeLa cells, TCTP inhibits autophagy through two separate pathways, 1. the AMPK/mTORC1 signalling pathway by inhibiting AMPK and activating mTORC1, and 2. through activation of Bcl-2, which in turn leads to an inhibition of the formation of the autophagic Beclin1 complex [53]. Both these studies demonstrated that TCTP is indeed able to modulate autophagy, however, the actual outcome is very much dependent on the precise cell physiologic conditions. Consistent with this, another paper on TCTP regulation of autophagy, published in this Special Issue, observed that TCTP negatively affects rapamycin-induced autophagy in the post-diauxic growth phase in yeast, but not autophagy induced by nitrogen starvation [54].

### 2.4. Extracellular Functions of TCTP(HRF)

Since the discovery of the histamine releasing factor (HRF) activity of TCTP in 1995 [55], it is established that, apart from its many intracellular functions, TCTP also acts as an extracellular molecule, typically in the context of immune reactions associated with allergic diseases. However, we will not expand on this, since the review by Kawakami et al. in this Special Issue [56] provided an up-to-date account on HRF and the current understanding of its involvement in various disease settings and its potential as a new therapeutic target. For a historic overview on the ‘HRF story’, the reader is referred to an earlier review article by Susan MacDonald [57].

## 3. Mechanisms of Regulation of Cellular TCTP Protein Levels

Being an anti-apoptotic protein involved in cellular stress responses, it is not surprising that the levels of TCTP are highly regulated. In our previous review article [5], we compiled a list of cell physiologic conditions that resulted in alterations of cellular TCTP levels. We also provided an overview on the principal mechanisms involved in TCTP regulation. As the name of the protein suggests, translational regulation plays an important role among these processes; it represents the most effective means for rapid de novo-synthesis. However, other modes of protein regulation, such as transcriptional control or regulated protein degradation, are also involved (Table 2, below). Since the publication of our review article [5], several new examples and mechanisms of regulation of TCTP levels have been revealed, and here we will largely focus on these more recent developments in this area.

### 3.1. Transcriptional Regulation

To date, numerous instances of transcriptional regulation of TCTP synthesis have been reported [5], but precise mechanistic investigations were only performed in a few cases. Thiele and coworkers demonstrated that TCTP synthesis is induced by phorbolester (PMA) through the cAMP-PKA signalling pathway and activation of transcription factor CREB [58]. They also studied the regulation of TCTP expression under stress conditions, such as heavy metals, and showed that copper induced TCTP synthesis at both the transcriptional and the translational level [59]. Several similar studies showed that TCTP is transcriptionally regulated under a variety of stress conditions [5].

Of particular relevance for cancer research was the demonstration that the tumor suppressor protein p53, acting as a transcription factor, negatively affects TCTP expression [40]. Since p53 is frequently mutated in cancers, this contributes to the observed deregulation of TCTP synthesis in cancers. A more recent paper reported another example of transcriptional regulation of TCTP expression [31]. The authors studied a specific transcription factor, insulin-response element binding protein 1 (IRE-BP1), whose decreased expression in insulin target tissues contributes to the development of insulin-resistant diabetes in rats. Normally, insulin-induced PI3K signalling results in the proteolytic cleavage of IRE-BP1 and release of the active C-terminal fragment of the protein. In order to study the target genes of IRE-BP1, the investigators analysed the proteome of pancreatic islets from transgenic mice overexpressing the active fragment of IRE-BP1, compared to the corresponding proteome from control mice. The overexpression of two of the identified target genes of IRE-BP1 in the transgenic mice was further confirmed by Western blotting, specifically the genes for RACK1 and for TCTP. Moreover, the authors discovered the interaction of RACK1 with TCTP by Co-IP experiments [31].

### 3.2. Translational Regulation

TCTP was originally discovered by the fact that its synthesis is induced after serum-stimulation of murine cells by translational activation of its preformed mRNA [1]. By 2016, several additional examples of translational regulation of TCTP synthesis were reported (reviewed in [5]), and among these, two principal translational control mechanism were found to be involved: (1) the growth factor-related induction of TCTP synthesis via the mTORC1-eIF4E signalling pathway [60,61], and (2) the negative regulation of TCTP synthesis via activation of PKR and eIF2α-phosphorylation. The latter occurs under serum-starvation [62] and also under Ca^2+^-stress conditions [63]. Our original observation that TCTP mRNA is a highly structured molecule that binds to and activates PKR [62] was further confirmed by a recent structural analysis of this mRNA under a various conditions, inclusive of the influence of riboSNitches [64].

Similarly, the regulation of TCTP synthesis through mTORC1 was recently observed in a very specific biological system, the retinal axon. Based on the findings from genome-wide screens, that TCTP mRNA is one of the most abundant mRNAs localised in the growth cone of axons, Roque et al. studied the role of TCTP in the development of the retinal axon in *Xenopus laevis*. They showed that it is indeed a protein essential for the normal development of the visual circuitry in the frog [18]. In a follow-up paper, the authors specifically investigated the translational regulation of TCTP and its importance for axon guidance [17]. They demonstrated that in axons of retinal ganglion cells, local TCTP synthesis is regulated by two axon guidance cues in an mTORC1-dependent fashion. In a completely different biological system, the intestinal stem cells in the midgut of *Drosophila*, yet another example of posttranscriptional regulation of TCTP synthesis was observed [15]. In this case, the Hippo signalling pathway has been implicated. However, the precise mechanism still awaits further characterisation.

The two translational control mechanisms referred to so far that target TCTP mRNA, act on the 5′-TOP (the mTORC1 signalling pathway), or on the highly structured area of the mRNA (for PKR activation). The latter most likely comprises the CG-rich 5′-UTR and a 5′-terminal stretch of the coding region [64]. Other translational control modes, targeting the 3′-UTR of TCTP mRNA, emerged only recently. This is surprising, since for 20 years, we have known that in many organisms, TCTP mRNA occurs in two isoforms, which differ in the length of their 3′-UTRs [65], with the shorter isoform usually being the most abundant one. Therefore, the biological importance of the second isoform is still elusive. In addition, for the regulation of TCTP expression in the axonal growth cone, it was shown that it is the shorter isoform, which is subject to translational regulation [17]. The first example of regulation of TCTP expression via the 3′-UTR of its mRNA was revealed in a recent, very interesting report [66]. The authors studied the expression of TCTP in *Trypanosoma brucei* and found that this unicellular parasite during its life cycle differentially expresses two TCTP paralogs, TCTP1 in the procyclic life form (in the tsetse fly) and TCTP2 in the blood stream form in humans. The two mRNAs largely differ in their 3′-UTRs, with TCTP2-mRNA bearing a 1.5-fold longer one. The authors demonstrated that the 3′-UTRs confer differential stability to these TCTP mRNA isoforms and that, for TCTP2 mRNA, the *cis*-acting element largely resides in the first 160 nucleotides of the 3′-UTR. However, the precise characterisation of the *cis*-acting element awaits further investigation.

Another mode of posttranscriptional regulation that targets individual mRNAs, typically via their 3′-UTRs, is the regulation by micro-RNAs. These short RNA molecules recruit ‘their’ target mRNAs to the RNA-induced silencing complex (RISC), which leads to translational arrest or even degradation of the mRNA. However, information about validated cases of TCTP mRNAs being regulated in this way is scarce (Table 2). In 2012, two papers reported that TCTP mRNA is regulated by miRNAs, i.e., miR-130a [67] and miR-27b [68], respectively. In the latter case, the authors observed that miR-27b levels were significantly reduced in tumor tissue of oral cancer patients, resulting in an increase of TCTP protein expression. Similarly, two recent papers reported on the deregulated TCTP expression in cancer, due to low levels of certain miRNAs, i.e., miR-145-5p in prolactinoma [69] and miR-125-3p in lung cancer [70]. In the latter paper, the authors studied the induction of TCTP in lung cells by tobacco smoke carcinogens. They found that miR-125-3p, is able to prevent the expression of a TCTP 3′-UTR reporter gene construct and that this miRNA is significantly down-regulated in xenografts generated from cells pretreated with these carcinogens.

### 3.3. Regulation of Protein Degradation

The mechanisms of regulated degradation of the TCTP protein were reviewed in detail in [34] and were also covered in a review article in this Special Issue in *Cells* [51]. Here, we will just briefly summarise the few cases known today (Table 2). Earlier papers reported the stabilisation of the TCTP (fortilin) protein by the anti-apoptotic protein Mcl-1 [71] and by the small heat shock protein Hsp27 [72], thereby preventing TCTP degradation in specific conditions, such as prostate cancer [73]. It was also shown that the antimalarial drug dihydroartemisinin (DHA), which is also used as an anti-cancer agent, binds TCTP and targets it for the ubiquitin-proteasome degradation pathway [74]. Another paper reported at the same time that, during mitosis and during meiotic exit, TCTP is partially degraded [75]. Our recent study characterised the pathway for specific TCTP degradation as acetylation-dependent chaperone-mediated autophagy (CMA), which eventually leads to lysosomal degradation of the protein and involves the proteins Hsc70 and LAMP-2A [76].

## 4. Disease Processes Involving Dysregulation of TCTP

### 4.1. Mechanisms of Cancer Promotion by TCTP

When we initially discovered that TCTP was overexpressed in a majority of tumors [77], it became clear that its inhibition could potentially result in decreased tumorigenicity. This reprogramming/reversion of cancer cells was found in breast tumor cells, lung cancer, colon carcinoma, and melanoma [77,78,79]. Typically, decreasing TCTP levels led to a restructuring of the tumor architecture, where breast cancer cells formed again ductal-like structures, reminiscent of normal tissues [77]. This suggested that TCTP regulates a series of oncogenic and tumor suppressor pathways and that its silencing suppresses malignant growth [79]. These aspects of TCTP have been extensively reviewed previously [78]. Since then, it has been confirmed that TCTP is overexpressed in most tumors including clinical samples [5]. The mechanistic way, through which TCTP exerts its action, is most probably by interacting with hundreds of proteins, influencing their function, in different ways [78,80].

As TCTP is a highly conserved protein expressed in all eukaryotic organisms, some of the crucial knowledge has been generated through work in *Drosophila* by K.W. Choi and colleagues. They found that TCTP regulates the TOR pathway through interaction with the dRheb GTPase [81]. Reduction of TCTP levels led to a reduced cell number, along with a smaller organ size. Given the importance of the mTOR pathway in cancer, these results provided one of the links between TCTP and tumorigenicity. These findings in *Drosophila* were further extended by showing the implication of 14-3-3 proteins; their interaction with TCTP and Rheb is necessary for the regulation of TOR [82]. The same group demonstrated that TCTP also regulates genome stability through modulation of dATM, one of the molecular complexes implicated in the DNA damage response [83]. In addition, they showed that TCTP binds to Brahma and negatively regulates its activity [84]. Brahma is the catalytic subunit of the SW1/SNF complex, which modulates chromatin and DNA repair, and which is mutated in more than 20% of human cancers [85]. In an elegant study from Azzam’s group [86], it was shown that TCTP forms complexes with ATM and γ-H2AX, suggesting a role in DNA damage and repair following exposure of human cells to low-dose γ-rays. Altogether, these data provide genetic evidence in support of the interaction of TCTP with the TOR-dependent oncogenic pathway, and of its role in maintaining genome stability.

Several studies link TCTP to epithelial to mesenchymal transition (EMT). This biological process is fundamental in early stages of embryonic development, such as the formation of the body plan during gastrulation [87]. Significant knowledge has been generated on the regulation of EMT in different developmental contexts, and evidence for its implication in cancer and metastasis is rapidly progressing [87], but still awaiting confirmation by clinical data. There has also been substantial progress in deciphering the molecular pathways involved in EMT and cancer [87]. The plasticity of cancer cells is key in EMT, especially in reprogramming somatic cells to ‘stemness’. Since TCTP plays an important role in tumor reprogramming, it was speculated that it might be part of the EMT induction process. In an interesting study [88], LLC-PK1 kidney epithelial cells were used to test in vitro the potential role of TCTP for the regulation of EMT. The overexpression of TCTP enhanced migration with a reduced expression of E-cadherin and increased expression of transcription factors repressing E-cadherin, such as ZEB1, slug, or twist. Depletion of TCTP reversed the EMT phenotype and suppressed migration. Results suggested that TCTP acts through metalloproteinases, specifically MMP-9, to facilitate cell invasion [88]. In addition, the study provided data indicating that TCTP regulates pulmonary metastasis of melanoma. Another study [89], this time using A549 lung adenocarcinoma cells, suggested that TCTP was a target of TGF-β1 and necessary for EMT and cytoskeleton reorganisation. Using the same cell line, it was further confirmed that TCTP promotes EMT and tumorigenicity [90,91] by influencing the expression levels of key transcription factors (ZEB1 and alpha SMA), and of miR-200a, miR-141, and miR-424 [90]. A very recent study also showed that TCTP is a key mediator in the induction of EMT by cigarette smoke carcinogens in lung epithelial and non-small-cell lung cancer cells [70]. Overall, these recent reports on the essential role of TCTP in EMT generated substantial support for our original observation [77] that decreased cellular levels of TCTP in cancer cells inhibit tumorigenicity by interfering with migration and invasion.

The capacity of TCTP to regulate diverse pathways, from mTOR and genome integrity to cell migration, has one common feature: cell survival. This is probably best reflected in tumor biology by reshaping tumor organisation and stemness [78], the latter being a conserved TCTP function from developmental biology [92] and tissue maintenance [15] to cancer stem cells [40]. A key aspect of TCTP function as a survival factor is its interaction with both, the anti-apoptotic and pro-apoptotic machinery. It was recently discovered that TCTP contains a BH3-domain, which is a common feature in the Bcl2 family regulators of apoptosis [93]. The crystal structure of a complex of Bcl-xL with a TCTP11–31 deletion variant revealed that TCTP refolds in a helical conformation upon binding the BH3-groove of Bcl-xL. Most importantly, TCTP potentiates the anti-apoptotic function of Bcl-xL, which is a unique feature [93]. On the other hand, TCTP has been shown to interact with the p53 tumor suppressor [40,78]. TCTP and p53 are involved in a negative reciprocal feedback loop, in which p53 represses the transcription of TCTP and the latter promotes the degradation of p53 [40]. This negative feedback loop is important for cell fate.

A study in hepatocellular cancer revealed that TCTP overexpression in this cancer induces mitotic defects [41], which is in line with other data showing that TCTP is involved in stabilising the mitotic spindle and is important for orderly mitotic/meiotic progression [94]. Our data also indicate that normal breast stem cells and cancer stem cells have an increased expression of TCTP [40]. As in developmental biology, stemness has to be protected from cell death. Importantly, we observed that breast cancer patients expressing high levels of TCTP in their tumors have a high grade, aggressive malignancy with a poor prognosis [40]. Similar observations have since been published for hepatocellular cancer (HCC) [41], colorectal cancer (CRC) [95], as well as for lung [90], breast [96], and gallbladder [97] cancer. Such elevated levels of TCTP in aggressive malignant disease may contribute to yet another problem, i.e., the enhanced resistance to various treatment modalities. For breast [98], lung [99], and colorectal [100] cancer cells, it has been shown that increased TCTP levels contribute to an increase in radio- and/or chemo-resistance.

Since the beginning of our research, it was obvious that TCTP is a potentially important target for cancer treatment. Sertraline and thioridazine are able to neutralise TCTP and hence, decrease its expression, which leads to apoptosis of cancer cells [79]. Sertraline is now being tested in phase I/II clinical studies [101]. Recently, it was demonstrated that TCTP is a promising target in melanoma, also using sertraline as a drug [102]. In our initial experiments targeting TCTP, we employed anti-histaminic drugs [79]. It has since been shown that anti-histaminic drugs interact with TCTP, and they were suggested as an approach to differentiation therapy [103]. The finding that the anti-malarial drug dihydroartemisinin (DHA), which also has anti-cancer activity [104], binds to and promotes the degradation of human TCTP (fortilin) [74], prompted initial studies to test the use of artemisinin derivatives against TCTP, either alone in gallbladder cancer [97] or in combination therapy against breast cancer [96,105]. Yet another approach, i.e., TCTP-antisense oligonucleotides, is being explored as a strategy against prostate cancer, and this seems to show some promise [106].

### 4.2. TCTP in Cardiovascular and Metabolic Diseases

Apart from cancer, TCTP dysregulation is also involved in a range of other disease processes (reviewed in [5,107]; for a compilation see Table 3 below). In a recent study, Cai and colleagues reported that TCTP plays a pivotal role in cardiomyocyte survival [46]. Downregulation of TCTP in cardiomyocytes induced cell death with apoptotic and autophagic features. Conversely, cardiomyocyte-specific overexpression of TCTP in mice resulted in decreased susceptibility to doxorubicin-induced cardiac dysfunction. In this case, TCTP acted as a disease-preventing factor. However, in the case of atherosclerosis, two earlier publications demonstrated that TCTP promotes the disease, albeit through two quite different mechanisms. Pinkaew et al. studied the effect of heterozygous deficiency of TCTP (fortilin) in mice, in a background of hypercholesteraemia [108]. They arrived at the conclusion that TCTP prevents apoptosis and therefore the reduction of macrophages, which are main contributors to the development of atherosclerosis. Kyunglim Lee’s group had previously shown that TCTP overexpression resulted in the inhibition of the Na,K-ATPase, and in the development of systemic hypertension in mice as early as six weeks after birth [109]. In a later paper, they demonstrated that, in ApoE-knockout mice, TCTP overexpression and consequently hypertension accelerated the development of atherosclerotic lesions caused by high-fat and high-cholesterol diet [110].

In a recent review article, K. Lee’s group summarised the consequences of the inhibition of the Na,K-ATPase by TCTP. Apart from the development of systemic hypertension, there are additional clinically relevant consequences, i.e., an increased tendency to form lens cataracts in mice and the activation of tumorigenic signalling pathways [111]. Another cardiovascular disease, in which a direct involvement of TCTP has been documented, is pulmonary arterial hypertension (PAH), a lethal disease caused by excessive proliferation of pulmonary endothelial cells (ECs). Using a proteomic approach, Lavoie et al. identified TCTP as one of 22 proteins that are significantly altered in the blood outgrowth ECs (BOECs) of patients with hereditary PAH [112]. Immunostaining revealed a marked increase in TCTP levels particularly in complex lesions of lungs from PAH patients, as well as in a rat model of severe and irreversible PAH. TCTP-knockdown led to an increase in apoptosis and to a reduction of the hyperproliferative phenotype in BOECs from PAH patients. In a recent follow-up study, the group also observed that silencing of TCTP in such BOECs resulted in significant alterations of the morphology and the migration behaviour of these cells [113]. They also demonstrated that TCTP can be transferred from ECs to pulmonary artery smooth muscle cells via exosomes, and in this way, the protein transfers the proliferative phenotype and apoptosis resistance onto neighbouring cell layers, thus playing a core role in the pathobiology of the disease.

Since TCTP is a cytoprotective protein involved in maintaining the cellular homeostasis of specialised cell types, its dysregulation may also play a role in metabolic disease states, such as diabetes. We found that in pancreatic β-cells, TCTP levels are regulated by glucose and that TCTP participates in protecting these cells against apoptosis induced by fatty acids [114]. In a more detailed study, Tsai et al. investigated the adaptation of β-cell mass in mice, both during early development and in insulin-resistant states, and found that TCTP expression correlated with phases of β-cell proliferation and mass expansion [115]. Specific knockout of TCTP in β-cells resulted in decreased growth signalling, β-cell proliferation and mass development, eventually leading to reduced insulin production and hyperglycemia. These observations received additional support by the recent finding that in mouse pancreatic islets, TCTP expression is regulated by the insulin-response element binding protein-1 (IRE-BP1) [31].

One of the pathologies associated with diabetes is nephrotic podocyte hypertrophy, which leads to an increase of glomeruli and to proteinuria. Kim et al. used a mouse model to study the involvement of TCTP in the development of this condition [116]. They found that TCTP knockdown reduced the activation of mTORC1 downstream effectors, the overproduction of cyclin-dependent kinases, as well as the size of podocytes and the glomeruli. In *db*/*db* mice, knockdown of TCTP prevented the development of diabetic nephropathy. Another type of hypertrophy, not related to diabetes, where TCTP overexpression was shown to be involved, is skeletal muscle hypertrophy. An in-depth study by Goodman and colleagues elucidated several aspects of TCTP’s role and regulation in skeletal muscle, using a range of mouse models [61]. They showed that TCTP is translationally up-regulated via the mTORC1 signalling pathway in skeletal muscle, under both hypertrophic and atrophic conditions. TCTP was sufficient to induce muscle fiber hypertrophy, and the protein may also be involved in inhibiting protein degradation.

Taken together, the various examples of diseases involving TCTP dysregulation (Table 3 below) show that, depending on the specific setting or cell type, the role of TCTP can be either in preventing or in promoting disease processes. TCTP as a cytoprotective protein may be involved in preventing the development of disease, as we see in model studies for cardiomyocytes [46] or pancreatic β-cells [115]. The properties of TCTP as a growth promoting and anti-apoptotic protein can also exacerbate disease processes, e.g., by driving cells into a hyperproliferative state, as in cancer (Section 4.1), PAH [112,113], diabetic nephropathy [116], or muscle hypertrophy [61], by preventing apoptosis, as shown for atherosclerosis [108] and PAH [112,113] or by inhibiting Na,K-ATPase, which leads to hypertension [111].

### 4.3. Allergic and Immune Disorders—TCTP as Histamine Releasing Factor

The discovery of the activity of TCTP as a histamine-releasing factor (HRF) has spurred a considerable research effort aimed at delineating its specific role in triggering cellular responses associated with allergic and other immune disorders. Various aspects of this work were reviewed on earlier occasions [5,57,117,118]; however, since then considerable progress has been made in understanding certain details of the role of TCTP/HRF in the development of various allergic disorders. These recent developments are summarised in a review by T. Kawakami and colleagues in this Special Issue in *Cells* [56], and for further details, the reader is referred to this article. Here, we will just mention a few core points: 1. After some initial controversy about the IgE-binding activity of HRF, it has been clarified that HRF binds to a subset of IgE molecules, in this way triggering histamine release [117]. 2. Several lines of evidence show that it is the dimer of TCTP/HRF, which is the active form for its extracellular activity [118]. The structure of the TCTP dimer has been solved and a model for dimerisation and the IgE binding site was derived from this [119]. A more recent paper also implied the flexible loop of the TCTP/HRF dimer in the activity of the molecule in triggering cytokine release from BEAS2B cells [120]. 3. The role and involvement of HRF in the following allergic disease states has been elucidated, at least in part, either in mouse models or in limited investigations in patients (Table 3): Asthma (reviewed in [56,117,118]), atopic dermatitis [121], food allergy [122,123], and chronic urticaria [124,125]. 4. A number of peptide and other inhibitors of TCTP/HRF showed some promise in alleviating symptoms elicited by this molecule in the context of allergic diseases [56].

Another paper published in this Special Issue of *Cells* reported quite a different aspect of TCTP’s involvement in inflammatory responses. The group of A. Senff-Ribeiro in Brazil had previously found that TCTP is part of the venom from the Brown Spider *Loxosceles intermedia*. They have now shown that TCTP is a synergistic factor contributing to the exacerbated inflammatory response elicited by the main toxin of the venom [126].

## 5. Synopsis

Recent years have seen a considerable deepening of our insight into the multiple biological functions of TCTP/fortilin/HRF, into the mechanisms of its regulation and into how dysregulation of the protein may contribute to various disease processes. At the cellular level, we learned more about the role of TCTP in the cell division process, both mitotic and meiotic; its function in stabilising polar spindle microtubules and the importance of the mitotic phosphorylation of TCTP by Plk1 for its detachment from the spindle. A novel mechanism for cell cycle control by TCTP was recently discovered in plants and insects. TCTP interacts with the CSN4 subunit of the COP9 signalosome complex to regulate cell cycle progression at the G1/S transition, through modification of the activity of Cullin–Ring ubiquitin ligases. This is important in both cell proliferation and organ development; however, the relevance of deregulation by this mechanism in diseases is yet to be documented. The importance of TCTP in organ development was further confirmed by additional examples in insects (tissue regeneration), in vertebrates (nervous system development) and in plants (lateral root formation).

The association of TCTP with the protein synthesis machinery was further consolidated by the discovery of its interaction with the additional ribosomal factors, EF1A2 and RACK1, as well as with mRNAs. Detailed structural studies confirmed that the previously reported binding of TCTP to EF1B is the most conserved interaction of TCTP. However, it is still an open question, whether TCTP affects protein synthesis rates generally, and/or modulates the translation of specific mRNAs. We certainly know several examples of TCTP regulating the stability of individual proteins.

The function of TCTP as a cytoprotective protein is well established, and several additional examples have now been reported. As a new mechanism, the involvement of TCTP in the unfolded protein response to prevent ER-stress was recently revealed. Autophagy is another important cell-homeostatic mechanism, and a few papers described the involvement of TCTP in this process as well, although the precise effect of TCTP on autophagy is still a matter of debate.

Cellular TCTP levels are highly regulated in response to alterations of a variety of cell physiologic conditions. We learned more about regulatory mechanisms that are involved in modulating TCTP levels. The list of transcription factors regulating TCTP mRNA synthesis has been extended by the tumor suppressor protein p53 and by IRE-BP1, an insulin-responsive transcription factor. We now know several translational control mechanisms, which may modulate the translational efficiency of TCTP mRNA; these are (1) signalling through the mTORC1 pathway, e.g., during growth induction of TCTP synthesis, (2) negative regulation by PKR in stress conditions, (3) mRNA stability regulation in Trypanosomes and (4) regulation through a small number of microRNAs in cancer. Several examples were reported, showing that TCTP levels may be modulated through regulated protein degradation. In serum starvation, TCTP was found to be degraded through chaperone-mediated autophagy.

The involvement of TCTP in cancer has been repeatedly proven. More examples of high TCTP levels being associated with a poor outcome in cancer patients have been reported. The participation of TCTP in the following cancer-promoting pathways has been demonstrated: the mTOR pathway and cell cycle progression, DNA repair and genome stability, antagonism to tumor suppressor p53 and anti-apoptotic activity, maintenance of ‘stemness’ in cancer cells, promotion of EMT and involvement in metastasis, and development of radio- and chemoresistance in cancer cells. Initial ideas to target TCTP as (part of) potential anti-cancer strategies have been published. Other disease processes, where dysregulation of TCTP might be a contributing factor, include cardiovascular diseases (arthrosclerosis and hypertension) and metabolic disorders (diabetes, muscle hypertrophy). The extracellular function of dimerised TCTP as histamine-releasing factor (HRF) in allergic and immune disorders has been further clarified, for asthma, atopic dermatitis, food allergy, and chronic urticaria.

With this extended knowledge about the principal functions of TCTP/fortilin/HRF in many biological and disease processes, our ‘toolbox’ should be large enough now for taking first steps towards ‘translating’ this knowledge into practical medical applications.

## Figures and Tables

**Table 1:** The importance of TCTP in basic biological processes.

Process	Examples	Potential Mechanism	References
Cell growth	Prevention of oocyte aging	Regulates spindle assembly	[12]
and organ	Cell cycle progression	Signalosome interaction	[13]
development	Insect development	Proliferation of epithelial cells	[14]
	Tissue regeneration	Stem cells; protein synthesis	[15,16]
	Brain development	(Axon guidance)	[18,19,20]
	Branching of plant roots	Initiation of lateral roots	[21]
Regulation	Association with proteins	(in yeast)	[22,23]
of protein	of the translational apparatus	(in human cells and *Drosophila*)	[7,15]
synthesis	- TCTP binding to EF1B	Regulation of the GEF activity	[24,25,28]
	- TCTP binding to EF1A2	of EF1B on EF1A	[7]
	- Interaction with RACK1		[31]
	- Binding to mRNAs	(Part of the mRNA interactome)	[32]
Regulation	Stabilisation of: HIF1α,	Prevention of	[34,39]
of protein	Mcl-1, PIM-3, PRX1, NTHK1	ubiquitination	[35,36,37,38]
degradation	Destabilisation of p53, Cdc25	Induction of ubiquitination	[40,41]
	Binding to proteasomes	Inhibition of proteasomes	[42,43]
Biological	Cardiomyocyte protection	Blocking apoptosis by Bnip3	[46]
stress	ER stress; (binding to IRE1α)	Blocking apoptosis by JNK	[47]
responses	Osmotic stress in plants	Increases photosynthesis	[48]
	Heat stress in Trypanosomes		[49]
Autophagy	Stimulation of autophagy	AMPK/mTORC1 pathway	[52]
	Reduction of autophagy	AMPK pathway; Beclin1	[53]
	Reduction of autophagy	Rapamycin-induced autophagy	[54]

**Table 2:** Mechanisms involved in regulating cellular TCTP levels.

Type of Regulation	Example	Mechanism Involved	References
Transcriptional	Induction by PMA	Transcription factor CREB	[58]
regulation	Copper stress reaction	Transcription and Translation	[59]
	Tumor suppressor p53	Inhibition of tpt1 transcription	[40]
	Insulin response	Transcription factor IRE-BP1	[31]
Translational	Induction of TCTP synthesis	via mTORC1-eIF4E signalling	[60]
regulation	- by growth stimuli	- in muscle	[61]
	- during axon guidance	- in retinal ganglion cells	[17]
	Negative regulation	Activation of PKR and	[62,63,64]
	in cell stress responses	phosphorylation of eIF2α	
	Developmental regulation	mRNA stability regulation	[66]
	in Trypanosomes	via the 3′-UTR	
	Regulation by micro-RNAs	miR-130a	[67]
	- in various cancers	miR-27b; miR-145-5p; miR-125-3p	[68,69,70]
Regulation	Stabilisation of TCTP	- by Mcl-1	[71]
of protein		- by Hsp27 (in prostate cancer)	[72,73]
degradation	DHA destabilises TCTP	Ubiquitin/proteasome pathway	[74]
	Partial degradation in mitosis	Ubiquitin/proteasome pathway	[75]
	Chaperone-mediated autophagy	Lysosomal degradation	[76]

**Table 3:** Dysregulation of TCTP in disease processes.

Type of Disease	Examples	Function of TCTP	References
Cancer	*Drosophila*; HCC	TOR pathway; cell cycle progr.	[41,81,82]
	*Drosophila*; human cells	DNA repair/genome stability	[83,84,86]
	Breast cancer	Antagonism to p53	[40,78]
	Various cancer cells	Anti-apoptotic activity	[40,78,93]
	Breast cancer	Maintaining cancer stem cells	[40,78]
	Lung cancer, melanoma	Promoting EMT	[70,88,89,90,91]
	Melanoma, gallbladder, CRC	Involvement in metastasis	[88,95,97]
	Breast, lung, CRC cells	Radio- and chemoresistance	[98,99,100]
Cardiovascular	Heart failure	Protection of cardiomyocytes	[46]
and metabolic	Atherosclerosis	- apoptosis in macrophages	[108]
diseases		- causing hypertension	[110]
	Hypertension, cataracts	Inhibition of Na,K-ATPase	[109,111]
	Pulmonary arterial	Proliferation, anti-apoptosis	
	hypertension (PAH)	in epithelial/endothelial cells	[112,113]
	Hyperglycemia, diabetes	Protects pancreatic β-cells	[114,115]
	Diabetic nephropathy	Promotes podocyte growth	[116]
	Muscle hypertrophy	Increase of TCTP and mTORC1	[61]
Allergic	Asthma	Dimeric HRF/TCTP binds to	[56,117,118]
and immune	Atopic dermatitis	IgE^+^ on mast cells/basophils	[121]
disorders	Food allergy	and triggers histamine	[122,123]
	Chronic urticaria	and cytokine release	[124,125]
